# Caring for a person with dementia during the COVID-19 pandemic: a qualitative study with family care-givers

**DOI:** 10.1017/S0144686X21000696

**Published:** 2021-04-29

**Authors:** Sabrina Cipolletta, Benedetta Morandini, Silvia Caterina Maria Tomaino

**Affiliations:** Department of General Psychology, University of Padua, Padua, Italy

**Keywords:** ageing, coronavirus, COVID-19, dementia, family care-giver, health psychology, pandemic, qualitative methods

## Abstract

The aim of this study was to explore the experience of family care-givers of people with dementia during the COVID-19 pandemic in the Veneto region of Italy to understand how and to what extent the emergency has affected care-givers’ lives and care routines. Twenty adult children of an ill person were interviewed via phone and video call, in adherence with the restrictions against COVID-19. Thematic analysis showed five main themes: the care-giver's experience, the care recipient's experience, relationships with care recipients, changes in the care routine and resources. Results pointed out that the time needed in the care routine and everyday activities increased during the pandemic, together with the need to find alternatives to physical activity at home. Depending on one's personal experience of COVID-19 and approach to preventive rules, the availability of resources, and formal and informal support, three main approaches to care were identified: apprehensive, mindful and fatalistic ones. The pandemic amplified the differences among these already-existing approaches to care as well as the typical challenges and difficulties experienced by family care-givers, and it resulted in an increased burden connected to practical difficulties, emotional stress and difficulties in reaching for help. These results underline the importance of strengthening the external support network for older people to help family care-givers, especially during emergencies.

## Introduction

The current coronavirus disease 2019 (COVID-19) is caused by a new coronavirus, which was first detected in December 2019 in Wuhan, China. The COVID-19 crisis was defined in March 2020 as a global pandemic by the World Health Organization (WHO, 2020*a*), a decision that urged governments to establish infection control measures such as social distancing, hygiene practices and lockdowns, thus imposing drastic changes in people's everyday routines and lifestyles (WHO, 2020*b*). Italy was the first Western country to be strongly affected by the pandemic and in which strict preventive measures were applied. There, the lockdown lasted from 8 March to 3 May 2020.

The first studies of the pandemic (Chew *et al*., 2020; C Wang *et al*., 2020) pointed out the importance of monitoring a population to comprehend the effects that the pandemic was having. Increased awareness of the emergency has been observed in the general population (Li *et al*., 2020; Lin *et al*., 2020), as well as anxiety, stress and vicarious traumatisation instilled by fear and media (Gao *et al*., 2020; Li *et al*., 2020; Odriozola-González *et al*., 2020; Rosen *et al*., 2020).

People with dementia, especially those who are older, more frail and at a severe stage of dementia, are at risk for different reasons: they are in a particularly risky age range to suffer the most severe consequences of the virus (Perrotta *et al*., 2020) and they are unable to understand or remember the preventive rules (Brown *et al*., 2020). COVID-19 tends to manifest differently in people with dementia, being more associated with such behavioural symptoms as delirium and worsening of functional abilities, with a diminished tendency to present usual symptoms such as dyspnoea and cough (Bianchetti *et al*., 2020). The COVID-19 emergency blocked social interaction and many other forms of stimulation (Brown *et al*., 2020; Leocani *et al*., 2020) that are essential for older people (Kuiper *et al*., 2015; Kelly *et al*., 2017). This situation heavily affected family care-givers, who were forced to change their care routine due to the prevention rules and to spend more time at home to protect their relatives (Brown *et al*., 2020).

Adult children of people with dementia normally experience distress, and especially those of a working age report experiencing many difficulties in maintaining a balance between work, housework, their care-giving role and, in some cases, their family and child care (Romero-Moreno *et al*., 2014). Moreover, they face the complexity of the course of dementia – of their parent gradually losing his or her ability to remember and communicate. Being a care-giver of a person with dementia imposes a greater burden than giving care to people with other diseases (Pinquart and Sörensen, 2003), due mostly to the experience of anticipatory grief (Holley and Mast, 2009). Other elements can increase the burden, such as the availability of other professional (Oliveira *et al*., 2019) and family (Yu *et al*., 2018) care-givers. Adult children of people with dementia tend to feel a strong sense of duty (Grigorovich *et al*., 2016) and guilt, connected to the feeling of not being helpful enough (Romero-Moreno *et al*., 2014).

In the specific period of the COVID-19 pandemic, people with dementia and their family care-givers have faced unprecedented and difficult challenges, especially in terms of a reorganisation of the daily routine and care relationship. In fact, adult children, as care-givers of people with dementia, have to balance different personal roles in their lives, being care-givers, workers and parents (Grundy and Henretta, 2006; Garcia-Ptacek *et al*., 2019). During the pandemic, some of the care-givers’ tasks conflicted with the preventive rules against COVID-19 diffusion (WHO, 2020*b*). Older adults need isolation to be protected, but at the same time, people with dementia need constant assistance and to be kept active (Wimo *et al*., 2018), and it is important further to keep such persons’ routines consistent (Alzheimer's Disease International, 2020). With the lockdown, all meeting places for people with dementia were closed, and it has become difficult to contact health-care services and associations for assistance (Brown *et al*., 2020). Experiencing practical difficulties in daily assistance and feelings of concern for the health of relatives, which is proven to have a negative impact on mental health, may characterise informal care-givers’ personal experience during the COVID-19 pandemic (H Wang *et al*., 2020). At the time this study was conducted, no study had explored this experience in care-givers of people with dementia.

Addressing the care role experienced by the care-giver, together with possible sources of fatigue, promptly and in a proper way, may lead to a better comprehension of care-giver needs and difficulties, making fostering the best support strategy easier. As pointed out by Cipolletta *et al*. (2018), family care-givers can be characterised by different levels of acceptance regarding their carer role: some may feel a strong sense of identity connected to their caring role, while others may experience caring as an unusual role for them, thus facing more difficulties in adapting and coping efficiently in the care relationship. In fact, family care-givers can experience fatigue, frustration and a consistent loss of time for themselves when caring for a loved one, and support offered to such care-givers may represent an opportunity to take time for themselves and to interrupt for a certain time the care routine in which they are immersed.

The aim of the present study was to explore and understand the experience of being a family care-giver of a person with dementia during the COVID-19 pandemic in one of the most affected regions in Italy and one of the first regions all over the world affected since COVID-19 spread out of China. This analysis may highlight the needs and difficulties family care-givers experience in implementing *ad hoc* support strategies.

## Method

A qualitative approach was implemented in our study as the gold standard to explore and deepen people's life experiences through a storytelling process that aimed for understanding rather than explaining, embracing rather than judging and remaining close to lived experience rather than analysing (Hurwitz *et al*., 2004).

### Participants

Twenty-one family care-givers of people with dementia were recruited. Inclusion criteria for participation were as follows: being the adult child of a person with dementia, being of working age, being an informal care-giver, living together with the parent with dementia or assisting them at their home, and both care-giver and parent being residents of the Veneto region of Italy. Participants were recruited by telephone by researchers thanks to collaboration with the Alzheimer Venezia Association, and then snowball sampling was used together with the publication of specific advertisements on thematic Facebook groups. Participation was voluntary and participants gave their informed consent to take part in the study.

One interview was excluded from the study because the interviewee was retired from work and thus did not match the inclusion criteria. Table 1 reports the personal data of the remaining 20 participants (17 females and three males) and their care recipients (13 females and eight males). The median age was 53 years, with a range from 42 to 67 years.

**Table 1.** Participants’ personal data

	N
Gender:	
Female	17
Male	3
Occupation:	
Working	4
Smart working	9
Layoff	2
Housewife	5
Marital status:	
Married	15
Single	4
Divorced	1
Children:	
No	5
Yes	15
Lives with the parents:	
Yes	7
No	12
Moved because of the emergency	1
Care recipient's diagnosis:	
Dementia (not specified)	9
Alzheimer's	2
Senile dementia	3
Frontotemporal dementia	1
Lewy body	2
Vascular dementia	2
Unknown	2
Period of the parent's illness:	
Less than 5 years	5
5 years or more	10
10 years or more	4
Unknown	2

*Note*: N = 20 care-givers, N = 21 care recipients.

### Measures

Semi-structured interviews (Brinkmann and Kvale, 2018) were performed between 8 April 2020 and 2 May 2020, a period in which Italy was in the strictest phase of its lockdown. Participants were interviewed via telephone or video calls to respect the anti-contagion measures. After a brief introduction to the interview, the interviewer read the information sheet and informed consent form; afterwards, participants had the chance to ask any questions of the researcher and gave their oral consent to participate in the study. Although conversational and flexible, the interview followed a common structure of open-ended questions, which was created *ad hoc* by the researchers based on existing evidence on family care-givers’ experiences and the research question that aimed to explore the specific experiences of care-givers in relation to the unprecedented situation caused by the COVID-19 pandemic. The main interview questions were the following: ‘Can you tell me something about the present emergency situation and how you are experiencing it? Has your typical daily life changed during the lockdown? Can you tell me how?’, ‘How do you feel about the fact that your parent is considered at the highest risk of infection? Are you adopting any particular precautions?’ and ‘How is your parent experiencing this situation? Have you noticed any changes in his/her behaviour lately? How do you interpret this change?’ The average duration of the interviews was 53 minutes (standard deviation = 22.7). Interviews were all audio-recorded and transcribed verbatim.

### Data analysis

A qualitative analysis of the interview transcripts was completed using thematic analysis (Braun and Clarke, 2012). This approach is intended to help understand the personal experience of participants by identifying recurrent themes in their narratives. Following an inductive bottom-up procedure with several recursive steps, two researchers identified and generated codes and themes from the interview data and not from *a priori* theory. Disagreements between the two coders were resolved through discussion with a third researcher. First, researchers became familiar with the participants’ narratives by reading the interview transcripts repeatedly and highlighting what was most significant. Second, initial codes, identified as recurrent in the data, were generated and linked to appropriate and exhaustive quotations and then organised into meaningful groups. Next, themes were clustered together to create superordinate themes and sub-themes. Moreover, the researchers read the interview transcripts again to verify that all salient themes had been correctly identified and that a persistent distinction was obtained between themes. Then, upon definition of a satisfactory thematic map of the data, themes were refined, named and organised into a coherent and internally consistent narrative. Last, the refined version of themes was embedded with exhaustive and verbatim quotations to support data discussion. The study was conducted according to the Consolidated Criteria for Reporting Qualitative Research checklist for reporting qualitative research (Tong *et al*., 2007). In accordance with the quality criteria for qualitative research (Yardley, 2017), coherence and reliability were achieved by accurately reporting the interviews’ administration, by data analysis and by the consistent use of each interview's quotations. The breadth and depth of the interviews enabled researchers to realise a comprehensive and extensive understanding of the experiences lived and reported by participants.

## Results

**Figure 1.**
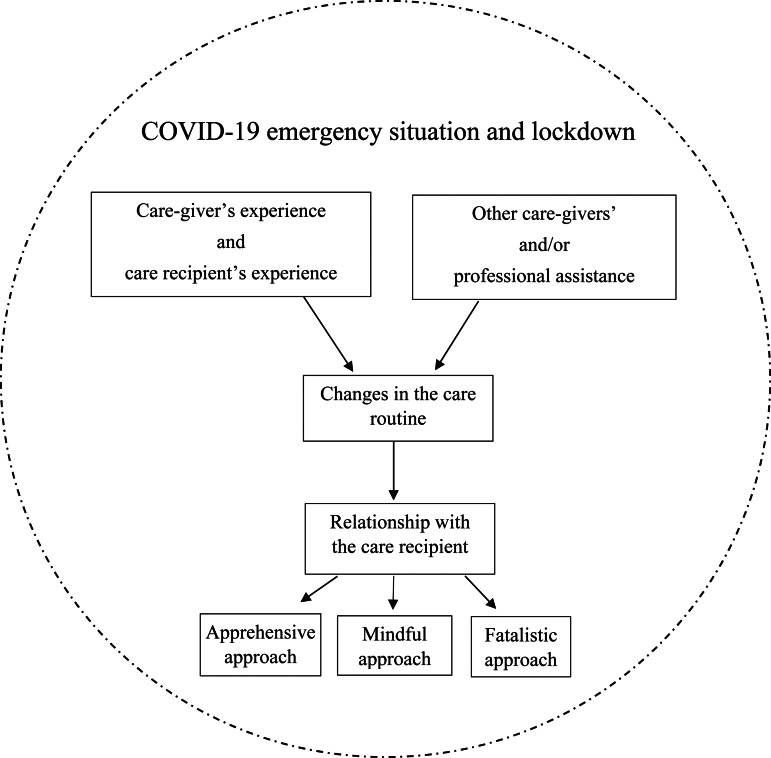
Map of the results.

Five main themes have been identified and listed in Table 2 with the respective codes. The relationships between themes are synthesised in Figure 1 and illustrated in the Discussion section.

**Table 2.** Themes and related codes

Themes	Codes
Care-giver's experience	• Lockdown experience • Concerns about COVID-19 • Nostalgia of social life • Economic difficulties • Concerns for the care recipient
Care recipient's experience	• Comprehension of the situation • Behavioural changes
Relationship with the care recipients	• Contact limitations • Dilemmas between keeping contact and respecting preventive measures • Video and phone calls
Changes in the care routine	• Grocery shopping • Prevention and attention to hygiene • Physical activity • Dedicated time to care-giving
Other resources	• Other family members’ support • Home care aides • Health-care system • Associations for people with dementia • General practitioners

### Care-givers’ experiences

During the lockdown, care-givers found themselves facing new and unknown experiences. Participants provided three interpretations of this period, viewing the lockdown as a positive, neutral or negative experience. Care-givers who described the lockdown as a positive time took the situation as an opportunity to spend more time with family, reporting positive changes in their routine in terms of less time dedicated to work and fewer obligations regarding the family's duties, connected to a sense of temporary relief and enjoyment. For some care-givers, the lockdown was a neutral experience, for they did not report any significant change in their normal routines in terms of familial or work duties. Care-givers who experienced the lockdown as a negative situation reported many events and routine tasks of daily life as highly stressful. The fear of infecting their loved ones, connected to an increasing demand of care and responsibilities for others during the lockdown, led to difficulties in finding free time for themselves and an increased perceived burden:

Let's say that right now it is even more difficult because we are always feeling the panic of transmitting her the virus. As soon as we see that she has a temperature or she is more agitated, then we immediately think, ‘It must be this [COVID-19].’ We are always in apprehension. (P5, female, 67)

One of the biggest concerns reported by care-givers was the possibility of infecting their older care recipients with COVID-19, and they mentioned being conscious of their care recipients’ vulnerability, fearing their death by the virus. In fact, some participants reported feeling very nervous and worried even if they were taking all precautions to protect their loved ones (wearing face masks and gloves) during the everyday care routine – even going outside their house – and were respecting lockdown measures as much as possible (*i.e.* going out only for groceries or necessities, limiting contact with others as much as possible):

The only fear that I have is to be or cause them harm, because anyway, I am conscious … that they take many medications and are absolutely the people at highest risk. (P21, female, 51)

Fear of infecting the care recipient made some care-givers avoid any external supports and move to the parent's house to assume exclusive responsibility for the care routine (usually administered by home care aides, nurses, formal care-givers, *etc*.). The media storm around COVID-19 generated even more panic and in some cases negatively influenced care-givers, leading to suppositions about some sort of manipulation by the government. Some care-givers reported nostalgia for their social life before the pandemic, especially in terms of being outside their house together with friends or relatives. They tried to compensate for this lack of social connection and routine activities through technological tools, but they did not feel particularly satisfied with online contacts compared to real-life ones. Economic difficulties were reported by care-givers; fears for their future stemming from lifestyle changes, economic crisis and unemployment were reported as highly stressful, especially in terms of possible negative consequences for the whole family and in the care process and activity with the care recipient:

It is not as before at all. I don't even know if it will ever be as before because my father's condition worsened and right now I am not working. I used to have a care aide to help me at home, and of course she is not coming right now, I don't even think I will be able to afford it [a care aide] in the future. (P21, female, 51)

All care-givers expressed fear of hospitalisation, especially for the care recipient, because it was associated with a risk of COVID-19 contagion and would result in long isolation and the impossibility of the care-giver visiting the parent. Awareness of their parent's fragile health and fear of death in the hospital were reported by care-givers as highly stressful and worrisome; they also expressed concern about the impossibility of organising a proper funeral:

Really, why should someone find himself dying in such conditions, without any relative by your side … with those nurses who are so blocked that you can't even see their faces, without having a direct sight, and you are there intubated in that chaotic situation that we all know well. (P20, female, 53)

### Care recipients’ experiences

Focusing on the care recipients’ experiences of the lockdown and the pandemic, care-givers reported that their comprehension of the situation was partial or non-existent for the whole period. Inability to comprehend was reported to be connected to the care recipient's Alzheimer stage and the severity of the disease. Major changes in the care routine, such as behaviours to respect isolation measures, wearing a face mask and gloves in and outside the house, and limiting movement outside the house and family meetings caused a sense of confusion in the care recipients:

[The care recipient asks] ‘Why are you wearing that mask?’ and I reply to her, ‘Because, Mum, there is a virus, and I could also infect you, so it is better to have my mask on’, and she asks me this every time, even five minutes later. (P7, female, 42)

In fact, care-givers reported mood changes and tension in their parents’ behaviour, caused by the perception of a generally tense atmosphere or nervous behaviour of the care-giver that negatively affected the care relationship. Frequent exposure to COVID-19 information via media communication and major changes in the everyday routine caused worries and confusion in the care recipients, resulting in frequent questions about the pandemic and care-givers’ preventive behaviours (wearing a face mask and gloves, maintaining physical distance, having no physical contact, *etc*.) being considered strange or unreasonable.

Care recipients in severe stages of dementia showed worsening behavioural changes in terms of ability to memorise, perception of time and presentation of more mood swings than usual. This symptomatology's worsening was attributed by family care-givers to the lockdown, the pandemic and changes in the care routine. Care recipients who showed worsening of symptoms were reported to be more demanding than usual, showing a major dependence on care-givers in terms of requests, time spent together, and need to be cognitively and physically stimulated, which resulted in a consistent reduction of care-givers’ free time:

I have seen a considerable worsening, let's say: he wants me always close to him, and obviously this means that I don't have any time for me. (P9, female, 59)

Only two care recipients were able to understand the situation fully and keep the pandemic and the preventive rules in mind, which led to the expression of personal suffering due to social isolation and distancing from family rather than a sense of confusion.

### Relationship with the care recipients

Care-givers reported difficulties in changing their way of physically interacting with a care recipient due to preventive rules and the lockdown. Face masks were described as ‘muzzles’ that block interactions and make it even more difficult to interact with care recipients. Care-givers reported missing the ability to interact freely and to help their parents with everyday activities, affirming that physical distance experienced first hand and with the rest of the family was having a negative impact on their relationship and the care recipients’ wellbeing:

The main difficulty that I feel for an older person with severe health issues is the obligation to keep her separated from loved ones, she must stay isolated … Relationships gave her life in some sense. (P4, female, 60)

Care-givers living with their parent or able to visit them experienced personal dilemmas between the benefit of the ability to stay in contact and the need to respect the rules for their safety.

In some cases, COVID-19 preventive rules affected interactions among families, care-givers and care recipients even more negatively. Care-givers who could not move to their parents’ house and were not co-habiting with them managed to keep in contact via phone or video calls. The possibility of making a phone or video call to their loved ones was reported as a benefit and a positive experience for the care recipients, although many difficulties were reported in the use of the technological devices and the identification of persons via calls. Video calls were reported as a tool to reunite families on special occasions, such as festivities or celebrations, thereby compensating for physical and social distancing:

Today, for example, it was the birthday of one of her sisters … We video-called her, and seeing each other made her [the care recipient] happy. Sometimes I video-call someone to let her see people; otherwise she would lose all social contacts, and they are already physically distant. (P13, female, 56)

### Changes in the care routine

To respect COVID-19 preventive rules and protect their parents, care-givers changed their care routines, mainly in four areas: grocery shopping, prevention and attention to hygiene, physical activity and time for the care recipient.

Five care-givers described grocery shopping as stressful, as the preventive rules lengthened the time needed for this activity and affected everyday time organisation.

Shops were reported as places where the risk of being infected was high, generating anxiety about infecting the care recipient once back home.

Care-givers expressed two approaches to preventive measures: one characterised by precision and strict respect of rules and the other less rigid, focused on care recipients’ needs and care-givers’ resources. Care-givers adopting the first approach strictly respected preventive rules (*i.e.* wearing a face mask and gloves, washing hands and wearing clean clothes when going to their parent's house), did not live together with the care recipients for the most part, received external support in caring for the parent and still went to work during the pandemic. These care-givers reported trying to convince the care recipients to respect preventive rules, such as wearing a mask and gloves, as a stressful action generally resulting in aggressive reactions or difficulties with understanding:

[The care recipient says] ‘Why should I wear this thing? What is it for?’ It is very difficult to make her understand that the mask is something to protect her health … She says, ‘I have always lived without one and nothing has ever happened – why should I change now?’ (P11, female, 58)

Care-givers adopting the second approach, especially those living with the care recipients, tended to respect only some of the preventive rules. The main difficulties in respecting rules were experienced especially in the context of the everyday care routine (while cleaning, eating and doing other activities), where maintaining distance and wearing a face mask were reported as virtually impossible for the care recipient and the care-giver:

On some occasions, it is also difficult to wear a mask; sometimes I eat with them, and at the table it is quite impossible to respect a one-metre distance and to eat with the mask on. (P7, female, 42)

Care recipients who lamented preventive measures or failed to comprehend them showed confused or aggressive behaviours towards the care-givers; thus, care-givers adopting this approach decided to lessen preventive rules inside the house to prevent confusion or discomfort of the care recipient. This lessening of the rules in the house did not correspond to a similar lessening outside; in fact, when going outside (*e.g.* for grocery shopping), precautions were respected to prevent being infected or infecting others.

Care-givers reported that the lockdown, with the impossibility of going out and engaging in physical activity, negatively influenced the care recipients’ motor functions – activity considered crucial for cognitive stimulation of the recipients. Only four care-givers were able to do some physical activity with the care recipient, such as short walks around the house in the open air (in a private garden or in the streets), respecting all precautions. This experience was reported as highly important to physically and cognitively stimulate the care recipient and to make both feel less trapped in the house during the lockdown:

Every day … we go out, we go for a walk with his walker and then he sits on it every four to five minutes. He sits on the walker, and I make him catch some sun. We sit in a place where we can see some people walking by; it is as if we are at a bar but with no bar. (P21, female, 51)

During the lockdown, increased time dedicated to care was registered as connected to the general lack of external support, the worsening of the care recipients’ symptoms and the increase of care necessities. Care-givers who mainly adapted their routine to their parents’ needs and rhythms during the lockdown experienced fatigue and a feeling of burden. By contrast, care-givers who dedicated less time to the care recipients’ needs and rhythms – due to work or family needs or not living together – kept in contact via telephone calls or socially distanced visits, reporting less fatigue but also distress connected to the fear of infection and separation.

### Other resources

In many cases, participants were not the only care-givers of the care recipients. The presence or absence of other care-givers (generally other family members) made a difference in terms of quality and quantity of care as well as wellbeing reported. The presence of other family members as active or potential care-givers of the care recipients was reported as an important resource in terms of practical support in the care routine and psychological support. Communicating with other people and receiving their help in the care routine reassured care-givers regarding shared responsibilities, time dedicated to care, and psychological support and understanding when experiencing burden:

[Referring to her husband and sons] However, if I ever feel sick they would certainly help me in caring for my parents. (P6, female, 59)

In some cases, the parent with dementia was not the only care recipient; other older adults in the family were also in need of care (spouse/husband, children and others), making the situation and the care routine more complicated for the care-giver. In many cases, due to preventive rules, other informal care-givers could not help with the care recipients during lockdown, causing stress and familial conflicts. The reasons were mainly to prevent infection and because of geographical distance:

My brother living in [location] used to come occasionally to help me. Unfortunately, when this emergency started, he couldn't come anymore. (P8, female, 47)

In those cases in which a home care aide used to assist the person, care-givers acted differently according to the situation: when the home care aide was not co-habitant, participants were recommended to move to the care recipient's house to limit the danger of infection. When they were already co-habitant, they asked home care aides to stay in the house most of the time to protect the care recipient. In some cases, home care aides were provisionally dismissed from their work because of the care-givers’ fear of infection. In fact, the impossibility of controlling home care aides’ behaviours and respect for preventive rules in the everyday care routine oriented care-givers toward preferring to bring their parents home for the lockdown period and manage care duties independently, generally leading to their dismissing home care aides. The management of home care aides during the emergency was reported as stressful and conflictual by many participants, but essential to handle the situation:

The only concern we have is related to the house care aide, because we don't really know what she does when she is not here. When she comes, we ask her to follow a sort of procedure; she must come in wearing gloves and a mask, and we ask her to clean deeply. To be sure, when she goes away, I clean the handles again. (P12, female, 45)

[The neurologist] told me: ‘You must decide what is the least bad option.’ (P1, female, 49)

A critical aspect reported was difficulty in the availability and accessibility of professional assistance during emergencies. All care-givers reported a lack of support and assistance by the national health-care system during the pandemic; this lack was experienced consistently before the pandemic as well, resulting in a general difficulty in seeking professional assistance when needed:

In case of need, it would be a great thing if there was some assistance. Especially for us [care-givers] who do not know how to behave and what to – yes, what to do in case of an accident or harm. Sometimes I ask myself, ‘If something happens, what should I do?’ and if she dies in such a peculiar time, so chaotic … It feels like there is so little support available. (P5, female, 67)

Care-givers reported referring mostly to local associations to find the support and assistance needed. In fact, the emergency slowed down bureaucratic procedures such as disability certificates, requests for specialised equipment and routine medical examinations, resulting in many practical difficulties and dissatisfaction.

Most participants referred to fear of hospitalisation for their parents, as they could become infected in hospitals; thus, they feared the possibility of their parents experiencing death in a hospital without family present or a funeral ceremony. As hospitals are no longer safe places for care, care-givers would rather be remotely supported by general practitioners or primary care services. Difficulties with receiving home visits by doctors, physiotherapists and other health-care professionals made care-givers feel abandoned and perceive disinterest in their fragile situations on the part of general practitioners, resulting in a general distrust and lack of reference points:

I don't know how to treat her [parent with Alzheimer's disease], I don't need doctors, I haven't found any health-care professionals that could help me; they seem uninterested in helping me. The only help provided is that I deliver myself or a close relative's support. (P12, female, 45)

Only nine participants reported having had the possibility to contact (through calls or emails) their general practitioners and geriatricians when needed during the lockdown. This contact reassured care-givers and fostered a sense of sympathy and understanding for health-care professionals in uncertain and difficult times:

The general practitioner is available to be contacted via telephone; fortunately, we didn't need to contact him until now … This period is difficult for everyone, and I think especially for doctors – for them, it must be even more difficult right now. (P15, female, 55)

Care-givers involved in associations for people with dementia before the pandemic reported that during the emergency, those associations kept in contact with people with dementia and care-givers, activating various forms of support for both. Tools and support were proposed, such as online self-help groups and materials (video, files, *etc*.) to stimulate care recipients, making the care-givers feel recognised and satisfied:

I am part of the Alzheimer Association. I took some courses with them and we have a WhatsApp group where they send us videos with examples of activities to be done in the house with the care recipients during the pandemic, as well as videos created by experts for us care-givers to give us some advice and reassurance. (P19, female, 46)

## Discussion

The results of the present study show that care-givers and care recipients could have different experiences of the COVID-19 pandemic and care process during the lockdown, resulting in different approaches to care. The emergency amplified the personal and professional challenges of care-giving for a person with dementia, in continuity with the pre-existing difficulties experienced in the care relationship and everyday life challenges of this specific population. A lack of consistent professional support and perception of abandonment, together with the negative impact of these experiences on the care relationship and state of wellbeing perceived by both care-giver and care recipient, clearly indicated the importance of addressing promptly risks and challenges enhanced by the pandemic.

### The three approaches to care

Three main approaches to care were identified, depending on personal experiences of the COVID-19 pandemic: approach to preventive rules, availability of resources, and formal and informal support. The first experience is characterised by apprehensiveness and is common among care-givers who were very worried about COVID-19 infection and the possible death of their care recipients; this made carers very attentive to their behaviours as a possible source of infection for their parents. These participants were worried about the lockdown and COVID-19, and often moved to the care recipients’ houses or moved the recipients to the care-givers’ houses to take care of them during the emergency. Home care aides, when present, were asked to stay home or dismissed from work because of the worries of infection and lack of control over them and their behaviour. These care-givers reported high levels of burden connected to the fact that strictly respecting preventive rules in and out of the house generated stress and discomfort for the care recipients and the care-givers, often resulting in aggressive or distressed behaviours and thereby influencing the care routine and relationship negatively. Personal stress, drastic changes in routine, a lack of free time, difficulties finding professional support during lockdown, and the stress and worries expressed by the care recipients emphasised the burden and distress experienced by the care-givers.

A mindful approach characterises care-givers who adopted a tailored method for personal and care recipients’ needs during lockdown and emergencies. They focused on needs, priorities and personal (as well as external) resources, trying to manage this new and difficult situation as best they could. Moving to their parents’ house or caring for them at the care-giver's home with a home care aide was a case-by-case decision based on evaluating pros and cons, dementia stage, the care recipients’ level of comprehension and external support. Preventive behaviours were respected to protect the care recipients, especially outside the house, but inside the house, they were adopted in a ‘flexible way’, with care-givers mindfully evaluating the situation and needs, and prioritising the comfort and tranquillity of the care recipients. In fact, as reported by care-givers, many people with dementia suffered physical and communicative limitations imposed by preventive rules, resulting in aggressive and discomfort behaviours that negatively influenced the care relationship and routine, dementia symptoms and care-givers’ wellbeing. These care-givers endeavoured to stimulate their parents physically and cognitively during lockdown, organising walks outside when possible and using exercises furnished by the associations they were in contact with before the start of the pandemic. To keep in contact with the care recipients (when not together) or facilitate the care recipients’ contact with other relatives, care-givers used video and phone calls, making them feel less lonely and isolated. When possible, other care-givers (formal and informal) were involved in the care process during lockdown to help and share responsibilities, which helped care-givers to feel less worried and decreased their perceived burdens.

A fatalistic approach characterises care-givers who minimise emergency situations. These participants believe that dementia itself is the worst illness and therefore do not fear COVID-19; they believe that care recipients are suffering already and if they should die from COVID-19 or another disease, nothing could be done to avoid it. These care-givers respected preventive behaviours outside the house, as it was mandatory to do so, but tended to underestimate their importance and were not concerned about the risks of infection inside the house and during the care routine. Care-givers had not changed their routine or lifestyle much since the start of the pandemic. The time dedicated to caring for the care recipients increased but did not require drastic changes in their routine. Family meetings or visits had not been suspended completely and were not reported as risky behaviours. Care recipients were reported as very fragile, mostly unable to understand the situation and lockdown as causing anxiety. A decrease in physical activity and less cognitive stimulation were reported as causes of worsening of behavioural symptoms connected to dementia, thereby causing conflicts as well as emotional and practical stress. Care-givers who adopted this approach reported a general sense of distrust and hopelessness related to the pandemic (and especially to dementia), together with anger and a sense of abandonment towards the health-care system and doctors. In fact, the impossibility of contacting them and the fear of hospitals as infected places influenced the care routine negatively for those care-givers and care recipients.

Similar approaches to care and care-giving have been explored in other studies of family care-givers of older people (Ehrlich *et al*., 2017) and people with different illnesses (Cipolletta *et al*., 2015, 2018, 2019). These results can be considered in light of the current studies on the effects of the COVID-19 pandemic. Okruszek *et al*. (2020) found that COVID-19 risk perception increases mental health symptoms by intensifying affective responses to the situation. As participants reported, lockdown and pandemic experiences negatively influenced their private lives, causing major anxiety and distress about the situation and personal and care relationships with the care recipients. Constantly fearing the infection and death of their parents, social isolation and worsening of distress behaviours resulted in an increased level of burden and excessive apprehension. In fact, constant fear of the care recipients’ death and self-blaming is a cause of burden and anxiety (Snyder *et al*., 2015; Liew *et al*., 2019). Prati (2020) found an association between constant and excessive concerns about the pandemic and burden; additionally, perceiving a higher risk and having less faith in preventive rules can affect mental health by increasing levels of anxiety, burnout and the need for psychological support (H Wang *et al*., 2020). The perception of self-efficacy plays a significant role in people's mental health; thus, the possibility of perceiving themselves as able to cope with the situation, together with the perception of efficient institutional support, enhances wellbeing and positive attitudes towards quarantine (Prati, 2020). Along with an increased level of burden connected to the pandemic, Milman *et al.* (2020) found that, for some, adhering to social isolation and other preventive measures may decrease anxiety.

### The amplification effect of the pandemic

Data from this study pointed out that the lockdown amplified the care recipients’ difficulties in comprehending the situation and caused aggressive behaviours, thereby resulting in an increased burden and fatigue for the care-givers. Receiving help from others could represent a risk of infection and was avoided by those participants who decided to manage the situation alone. These care-givers experienced more burden and had no free time. Being aware of sacrificing personal time and feeling a strong sense of duty may cause higher levels of burden (Grigorovich *et al*., 2016; Garcia-Ptacek *et al*., 2019). On the contrary, social support may alleviate the burden of care from a practical and psychological point of view, because it allows the care-giver to confide in someone else who can take on the care-giver's role in case of need (Cipolletta *et al*., 2014, 2018, 2019; Brand *et al*., 2016). In fact, as Pancani *et al.* (2020) pointed out, offline and online social contacts could mediate the adverse effects of social restrictions. As our participants noted, social connection was reported as a source of vital energy for care recipients and a source of practical and psychological support for care-givers themselves.

The lockdown, as an unknown and unfamiliar experience, amplified existing problems. These issues included the need to equilibrate family, work and care-giving (Romero-Moreno *et al*., 2014); the need to maintain stable routines for people with dementia (Alzheimer's Disease International, 2020); a lack of free time (Romero-Moreno *et al*. 2014; Grigorovich *et al*., 2016) and increased care time needed; the need to find personal resources, such as coping strategies, to deal with the difficulties of caring for a person with dementia (Snyder *et al*., 2015; Frota Montiero *et al*., 2018); lockdown difficulties such as fear of infection and social isolation, and difficulties in receiving institutional and external support (Chew *et al*., 2020).

The COVID-19 emergency amplified the sense of abandonment and isolation due to the poor availability of and accessibility to formal support (health-care services, general practitioners, *etc*.) during the pandemic, which, participants reported, was also experienced before the emergency. Local associations were considered useful resources for care-givers and care recipients during the emergency. As reported by the literature (Reuben *et al*., 2019), comprehensive care programmes working with care recipients and care-givers demonstrated clinical benefits for both and facilitated interactions between the two, resulting in the maintenance of a low burden level for care-givers. As our participants confirmed, support activities offered by health-care services succeeded in facilitating interactions between care-givers and care recipients by providing practical opportunities (exercises and tasks) for care-givers to stimulate care recipients and making the care-givers feel considered and validated.

As our participants reported, lockdown and social isolation increased the need for a specific support network able to assist care-givers and care recipients in their needs, especially in cases of emergency (Siegrist and Zingg, 2013). The impossibility of keeping in contact with health-care professionals and external support during lockdown mainly influenced the perceived burden and distress care-givers reported. As informal care-giving has increased over the years (Ma *et al*., 2018), it is crucial to move forward in the development of specific support resources to assist care-givers and families in emergency moments as well as everyday activities. A strong network among professionals is vital to help families face this delicate and demanding task.

### Limitations

The main limitation of this study was the use of phone and video calls for the interviews, which posed technical problems for some care-givers and limited the richness of expression of some experiences. Moreover, this study took place in a region that managed the infection optimally in terms of health-care response; thus, it is possible that the psychological impact of the emergency was perceived less negatively than in other regions.

## Implications

It has been pointed out that high levels of burden and distress together with care-givers’ personal coping strategies can negatively influence people with dementia (Garcia-Alberca *et al*., 2013; Norton *et al*., 2014; H Wang *et al*., 2020) during a pandemic (Taylor, 2019). Furthermore, fewer social interactions and limitations to physical activities can damage those with dementia (Kuiper *et al*., 2015; Kelly *et al*., 2017). As reported by other studies (Fernandez *et al*., 2002; Ford *et al*., 2006; Aldrich and Benson, 2008; Christensen, 2012), the need to monitor older people during emergencies is crucial because of their vulnerabilities, which is why it is necessary to activate a more solid network. Furthermore, differentiating within caring experiences and identifying different care approaches may be useful to construct personalised support strategies for care-givers and care recipients in emergencies. More specifically, for those with a mindful approach, offering information and more resources may be sufficient to support their caring activities. Care-givers experiencing higher levels of worry and apprehension may be supported through reassurance, but also by promoting their trust in care recipients to help them encourage the autonomy and wellbeing of their loved ones, resulting in the enhancement of their personal wellbeing and a reduction of burden. Finally, those with a more fatalistic approach may be encouraged to attend to preventive measures more strictly by facilitation of their understanding that improved coping with the COVID-19 pandemic may help them and their loved ones to cope better with dementia as well. Further studies are needed to explore these strategies.

The findings of this study pointed out the importance of designing innovative care resources for this specific population, especially in times of health crises and social isolation, where Italian families are in charge of caring for older parents with severe conditions. In Italy, 79 per cent of family care-givers are female (McKee *et al*., 2003), and daughters in particular are expected to care for older parents at home. Care is perceived as a duty in terms of returning the care and attention they received as children, thus resulting in conflicting emotions, guilt, psychological fatigue, and difficulty balancing work- and family-related responsibilities (Chio *et al*., 2005). As many participants reported, families and care-givers feel unprepared and abandoned when caring for a person with dementia, and the specific situation of a global pandemic enhanced existing difficulties and burdens, pointing out the need for a more prompt and tailored intervention programme and a range of support resources.

In COVID-19 times, the impossibility of getting help at hospitals or clinics has emphasised the relevance of technological modalities and resources in supporting connections and collaboration between health professionals, care-givers and people with dementia. Supporting from afar with consistency – thus filling the gap of the Italian health-care system that was deployed on the front lines against the pandemic – might have helped care-givers and care recipients deal with such uncertain times and unexpected difficulties.

## Conclusion

The COVID-19 pandemic and lockdown posed new challenges in care relationships and routines of people with dementia and their family care-givers.

Family care-givers’ personal experiences of the pandemic and lockdown, their attitudes towards prevention rules, their relationships with the care recipients, different levels of burden experienced, and the presence or absence of other care-givers impacted the care recipients’ wellbeing and the care relationship, resulting in three care approaches. Moreover, advanced stages of people's illness, a lack of understanding of the situation and the preventive measures, and a sense of abandonment on the part of the health-care system and support network increased care-givers’ fatigue and burden.

The care approaches identified in this study can be understood in light of the pandemic situation and thus as adaptive strategies care-givers used in response to the amplification of dementia challenges as well as difficulties experienced by both care-givers and care recipients. These approaches to care, though distinct from each other, together underline the urgency of innovating and expanding the support and care programmes offered by health-care services, especially on a local level. Furthermore, being able to identify different adaptive strategies and care approaches in an emergency situation (COVID-19 or otherwise) will enable health-care professionals and services to address difficulties and challenges properly and promptly for care-givers and care recipients. This will help prevent the amplification of burden for the care-giver and the worsening of symptoms for people with dementia, as well as strengthening care-givers’ coping strategies and access to professional support networks.
